# Sex based differences in functional connectivity during a working memory task: an fNIRS study

**DOI:** 10.3389/fpsyg.2024.1207202

**Published:** 2024-02-08

**Authors:** Sima Shirzadi, Mehrdad Dadgostar, Zahra Einalou, Sinem Burcu Erdoğan, Ata Akin

**Affiliations:** ^1^Department of Biomedical Engineering, Central Tehran Branch, Islamic Azad University, Tehran, Iran; ^2^Athinoula A. Martinos Center for Biomedical Imaging, Massachusetts General Hospital, Harvard Medical School, Charlestown, MA, United States; ^3^Department of Biomedical Engineering, Acibadem Mehmet Ali Aydinlar University, Istanbul, Türkiye

**Keywords:** sex differences, fNIRS, functional connectivity, working memory, wavelet transform, partial correlation

## Abstract

Differences in corticocerebral structure and function between males and females and their effects on behavior and the prevalence of various neuropsychiatric disorders have been considered as a fundamental topic in various fields of neuroscience. Recent studies on working memory (WM) reported the impact of sex on brain connectivity patterns, which reflect the important role of functional connectivity in the sex topic. Working memory, one of the most important cognitive tasks performed by regions of the PFC, can provide evidence regarding the presence of a difference between males and females. The present study aimed to assess sex differences in brain functional connectivity during working memory-related tasks by using functional near-infrared spectroscopy (fNIRS). In this regard, nine males and nine females completed a dual n-back working memory task with two target inputs of color and location stimuli in three difficulty levels (*n* = 0, 1, 2). Functional connectivity matrices were extracted for each subject for each memory load level. Females made less errors than males while spending more time performing the task for all workload levels except in 0-back related to the color stimulus, where the reaction time of females was shorter than males. The results of functional connectivity reveal the inverse behavior of two hemispheres at different memory workload levels between males and females. In the left hemisphere, males exhibited stronger connectivity compared to the females, while stronger connectivity was observed in the females' right hemisphere. Furthermore, an inverse trend was detected in the channel pairs with significant connectivity in the right hemisphere of males (falling) and females (rising) by enhancing working memory load level. Considering both behavioral and functional results for two sexes demonstrated a better performance in females due to the more effective use of the brain. The results indicate that sex affects functional connectivity between different areas in both hemispheres of the brain during cognitive tasks of varying difficulty levels although the general impression is that spatial capabilities are considered as a performance of the brain's right hemisphere. These results reinforce the presence of a sex effect in the functional imaging studies of hemodynamic function and emphasize the importance of evaluating brain network connectivity for achieving a better scientific understanding of sex differences.

## 1 Introduction

Investigation of sex dependent differences in neurological functions has been an interesting area of research for neuroscientists. Several neuroimaging studies (Kameyama et al., 2004; Tomasi and Volkow, 2012; Chuang and Sun, 2014) have examined the presence or absence of structural and functional differences between the brains of males and females. Results from these studies showed that males and females' brain have differences in functional organization, for example, recruiting different brain regions or the amount of activation in each hemisphere and brain areas both at the rest and during different cognitive tasks (Speck et al., 2000; Bell et al., 2006; Tüdös et al., 2018). Objective and accurate quantification of these functional differences may allow early diagnosis, more accurate treatment, and better disease management especially for neuropsychological disorders such as ASD (autism spectrum disorder) and schizophrenia, which are associated with changes in structural and functional connectivity in the brain. In addition, the roots of many behavioral differences between sexes can be traced from a neurological perspective (Kimura, 1992; Cahill, 2006).

Despite commonalities in functional and anatomical organization of human brains across sexes, the brain of each individual has a unique local connectome, like a fingerprint (Finn et al., 2015). Cognitive performance is closely linked to how brain regions are functionally and structurally connected and how these connections vary among individuals. Differences in cognitive as well as functional and structural connectivity have been suggested to be mainly determined by genetic, hormonal and environmental factors, and may form the basis for regulating human behavior and reactions to environmental stimuli (Cosgrove et al., 2007; Zaidi, 2010).

Psychological differences among males and females have been thoroughly investigated since the advent of psychology in 1879 (Shields, 1975). Although differences in behavioral and emotional reactions of males and females may seem to be related to culture, progressions of recent studies indicated that many questionable sex differences are rooted in the specializations of the human brain for processing different types of stimuli.

The brains of males and females represent anatomical, functional and biochemical differences at all life stages. Several studies reported the significant effect of sex on many human cognitive performances such as perception, emotions, language, intellectual problem-solving, memory, and other cognitive domains (Cahill, 2006). Males and females use their two brain hemispheres differently in some of these cognitive performances (Kimura, 1992; Gur et al., 1995; Shaywitz et al., 1995). Since the brain is responsible for controlling and regulating cognition and behaviors, these functional differences between males and females may be related to the sex-specific evolution of the structure of brain network connectivity and topology. Consequently, the evaluation of differences in neural performance during processing different types of cognitive stimuli may be useful for understanding the topological processes involved in neural (Poudel et al., 2015) and psychiatric disorders (Henseler et al., 2010). Based on the results of the assessments from the perspective of gland physiology, the brain is considered as an important target for gonadal steroid hormones (McEwen et al., 1984). Due to the sensitivity of the prefrontal cortex (PFC) to the fluctuations in gonadal hormones, these hormones may influence neural organization at the microscopic level, as well as cognitive functions and the relative output performance. PFC has estrogen receptors, and estrogen regulates the activity of certain neurotransmitter systems in the PFC. When these receptors are activated, synaptic transmission, neurogenesis, and neuroprotection may change. It can improve synaptic plasticity and promote the formation of new neuronal connections, which can affect cognitive processes such as working memory (WM). For example, increasing estrogen level can improve PFC-related performances such as working memory (Duff and Hampson, 2000; Cosgrove et al., 2007). Based on Baddeley's theory, working memory is considered as a temporary buffer for storing and maintaining information actively, along with their manipulation, while it plays a fundamental role in many cognitive performances such as mental calculations, reasoning, language perception, and spatial capabilities (Baddeley, 1992; Owen et al., 2005). Working memory can provide evidence on the existence of differences among males and females since it is a central performance of PFC. In general, while some studies have shown no performance differences in behavior between sexes, other studies have demonstrated that males and females have different behavioral performance in various tasks. Therefore, further investigations in this area, considering various factors involved in behavioral performance, are needed to achieve more reliable evidence in this field. In recent years, various imaging techniques have been presented for assessing how the brain performs during cognitive tasks, including positron emission tomography (PET), electroencephalography (EEG), diffusion tensor imaging (DTI), magnetoencephalography (MEG), and functional magnetic resonance imaging (fMRI). However, these imaging systems are expensive and possess a high sensitivity to motor artifacts. fNIRS is an optical and wearable brain imaging technology which can monitor and parameterize local hemodynamic changes induced by cortical brain activation in naturalistic settings. This method derives the relationship between metabolic activity due to neural processing with effectively measured temporal changes in the concentration of oxygenated hemoglobin (HBO) and deoxygenated hemoglobin (HBR) in blood vessels. fNIRS measures regional hemodynamic changes associated with cortical activity by using this strong and close relationship between neuronal activity and regional blood flow in the brain (Villringer and Dirnagl, 1995). In recent years, this method has been highlighted as a non-invasive technique for the functional neuroimaging of the brain. Also, its low cost and high portability allow examining in a natural environment and have the major advantages of being less restrictive and less sensitive to motion artifacts when compared to EEG, while providing a better spatial resolution. Thus, it is suitable for measuring brain functional activity in patients with mental and neural disorders (Fallgatter et al., 1997; Matsuo et al., 2002; Suto et al., 2004).

In a large body of research, fMRI was applied in various verbal working memory (verbal WM) and spatial working memory (spatial WM) tasks since its emergence as a non-invasive method for detecting brain activity during different tasks (Smith and Jonides, 1999). Furthermore, the differences among males and females were reported in some activation studies by using fMRI. The lateralization of word processing was one of the first findings on neurofunctional differences between sexes (Shaywitz et al., 1995). Word processing is associated with activation of the left-sided Broca's area in males, as well as its bilateral activation in females (Rossell et al., 2002). Numerous fMRI studies referred to the sex differences in the structural and functional characteristics of the human brain (Zaidi, 2010). An fMRI study conducted by Tomasi and Volkow (2012) found that females have 14% more local functional connectivity density (lFCD) and 5% greater gray matter density in cortical and subcortical areas compared to males. The sex differences in working memory were evaluated through various tasks such as letter, face, word, and number n-back (Speck et al., 2000; Haut and Barch, 2006; Schmidt et al., 2009), auditory Q3A-INT (Goldstein et al., 2005), and number recognition in some fMRI studies (Bell et al., 2006).

Based on the evidence, different working memory tasks (verbal WM and spatial WM) utilize various brain networks (Na et al., 2000). Further, the results of recent working memory studies demonstrated the impact of sex on organizing brain connectivity patterns, which indicates the important role of connectivity in sex topics.

During recent decades, several studies have been conducted by using fNIRS to evaluate the neural resources employed in working memory tasks. These studies examined the effects of memory load on the activation and connectivity of different brain areas through various tasks. Li et al. assessed the males-females differences in PFC performance during the n-back verbal memory task by using NIRS and recorded the changes in the concentration of oxyhemoglobin, deoxyhemoglobin, and total hemoglobin (Li et al., 2010). They reported obvious differences in oxyhemoglobin and total hemoglobin among males and females, as well as no difference in the deoxyhemoglobin. Also, males exhibited bilateral activity with a slight dominance of the left hemisphere, while females represented left-hemisphere activation. Males's brain activity localization was wider and the magnitude was stronger than that of females. Furthermore, females needed a lower hemodynamic supply for obtaining a performance comparable with that of males, and a positive correlation was attained between hemodynamic parameters and behavioral performance only among females. The results reinforced the existence of sex effect in the hemodynamic-based functional imaging studies. Indeed, the results of their study indicated higher efficient hemodynamics in PFC during working memory tasks among females and emphasized the importance of evaluating PFC for attaining a greater scientific understanding of males-females differences. Despite the similarities between sexes regarding behavioral performance, sex differences were observed in PFC hemodynamic responses.

Several studies have examined the behavioral differences among males and females in various working memory tasks, some of which reflected that males are better than females in 2D and 3D mental rotation tasks (Kaufman, 2007; Silverman et al., 2007; Nori et al., 2012). However, focusing on the spatial WM resulted in achieving contradictory results about males-females differences, which were measured through the Corsi Block-Tapping (CBT) task (Corsi, 1972). The results of various studies indicated a higher memory span in males compared to females (Orsini et al., 1987; Piccardi et al., 2013), while no difference was found among the sexes in other studies (Smirni et al., 1983; Nichelli et al., 2001; Farrell Pagulayan et al., 2006). Furthermore, the sex differences were examined by conducting a study using verbal WM and visuospatial working memory tasks with and without distraction conditions in the intervals of the tasks. Based on the results of the study, no significant difference was observed in word recalling and verbal WM without distraction conditions among the sexes. During the task with distraction, females were weak in recalling verbal stimuli compared to males, while they recalled stimulus significantly better in the visuospatial working memory task (Harness et al., 2008). In another study, the letter n-back task was applied for examining the males-females differences in working memory performance. Based on the results, no significant difference was achieved regarding sensitivity to targets among the sexes in the task (Evans and Hampson, 2015). Table 1 provides a summary of studies conducted to examine sex differences using neuroimaging techniques.

**Table 1:** A short summary of different neuroimaging techniques used to study sex differences.

**Sex differences in some neuroimaging studies**		
**Technique**	**Task**	**Result**
				***Males/Females***
fMRI (Tomasi and Volkow, 2012)	Resting-State	The negative power scaling of the local functional connectivity density was steeper in men. There is no significant difference in functional connectivity density or brain activation between the sexes.
fMRI (Speck et al., 2000)	Verbal Working Memory	Bilateral activation or right-sided dominance (LPFC, PC and caudate) Predominant activation in the left hemisphere
fMRI (Shaywitz et al., 1995)	Phonological Task	Lateralized brain activation to the left inferior frontal gyrus regions in men The pattern of activation involved both the left and right inferior frontal gyrus in women.
fMRI (Goldstein et al., 2005)	Auditory Verbal Working Memory	Left-sided activation of Broca's area in males Greater activation in prefrontal regions and Bilateral activation of Broca's area in females
fMRI (Rossell et al., 2002)	Word Processing	Left-sided activation of Broca's area in males Bilateral activation in females
fMRI (Tian et al., 2011)	Resting-State	Males were more locally efficient in their right hemispheric networks, but females were in their left hemispheric networks.
EEG, EOG (Shen, 2005)	Stroop Task	The P300 latency was longer and the amplitude was smaller in males than females.
NIRS, EEG (Gao et al., 2018)	Verbal Sternberg Tasks	Males need more brain activation as well as more optimized brain networks. Females surpass men in verbal WM from the perspective of both brain activation and connectivity.
NIRS (Li et al., 2010)	N-back Verbal Working Memory	Bilateral activity with a slight dominance of the left hemisphere and a stronger magnitude in males Left-hemisphere activation in females

Working memory is considered as responsible for storing information actively, along with their updating, and directing individuals' performance during tasks requires the continuous use of the changing information. Accordingly, heavy demand is created on the working memory system. Since males and females adopt various strategies for information encryption (Gao et al., 2018), it is not unexpected to observe the sex differences in brain connectivity during working memory-related tasks.

The innovation of the present study was the use of fNIRS signals during a 2D spatial WM task for evaluating the sex differences in the functional connectivity of the frontal cortex. To this end, a dual n-back working memory task with two target inputs of color and location stimuli was performed at three difficulty levels (*n* = 0, 1, 2) among two groups of males and females. Then, brain functional connectivity was extracted by using the partial correlation coefficient (PCC) which was obtained between denoised signals of different brain regions. Finally, the differences in the connectivity between various memory load levels were examined through statistical tests among males and females.

## 2 Materials and methods

### 2.1 Subjects

In the study, 18 healthy right-handed volunteers participated, among whom nine were males (mean age = 24.2 ±4.58) and the others were females (mean age = 24 ± 4.49). The individuals were considered physically and mentally healthy, as well as no history of a neural disease or mental disorders, and taking certain medications. All of the subjects possessed normal color and correct vision and provided an informed consent form before task evaluation. This research was approved by the Ethics Committee of the Iran University of Medical Sciences (Code: IR.IUMS.REC. 1397.161).

### 2.2 Task procedure

The n-back, introduced by Kirchner (1958), is considered as one of the most common and widely-applied tasks for assessing working memory capacity and capabilities. This continuous performance task is often used in neuroimaging for stimulating individuals' brain activity. In the n-back task, subjects observe or hear a sequence of stimuli and respond when a target is presented. However, the target is constantly changing and depends on previous stimuli so that the task becomes more difficult by increasing memory workload level (n).

Jaeggi suggested the dual n-back task (Jaeggi et al., 2003), in which two independent sequences of stimulus (the auditory and visual stimuli) are presented at the same time. These stimuli are usually different such as auditory and visual stimulation. Then, subjects should control two possible targets in each trial, which makes the task difficult.

**Figure 1:**
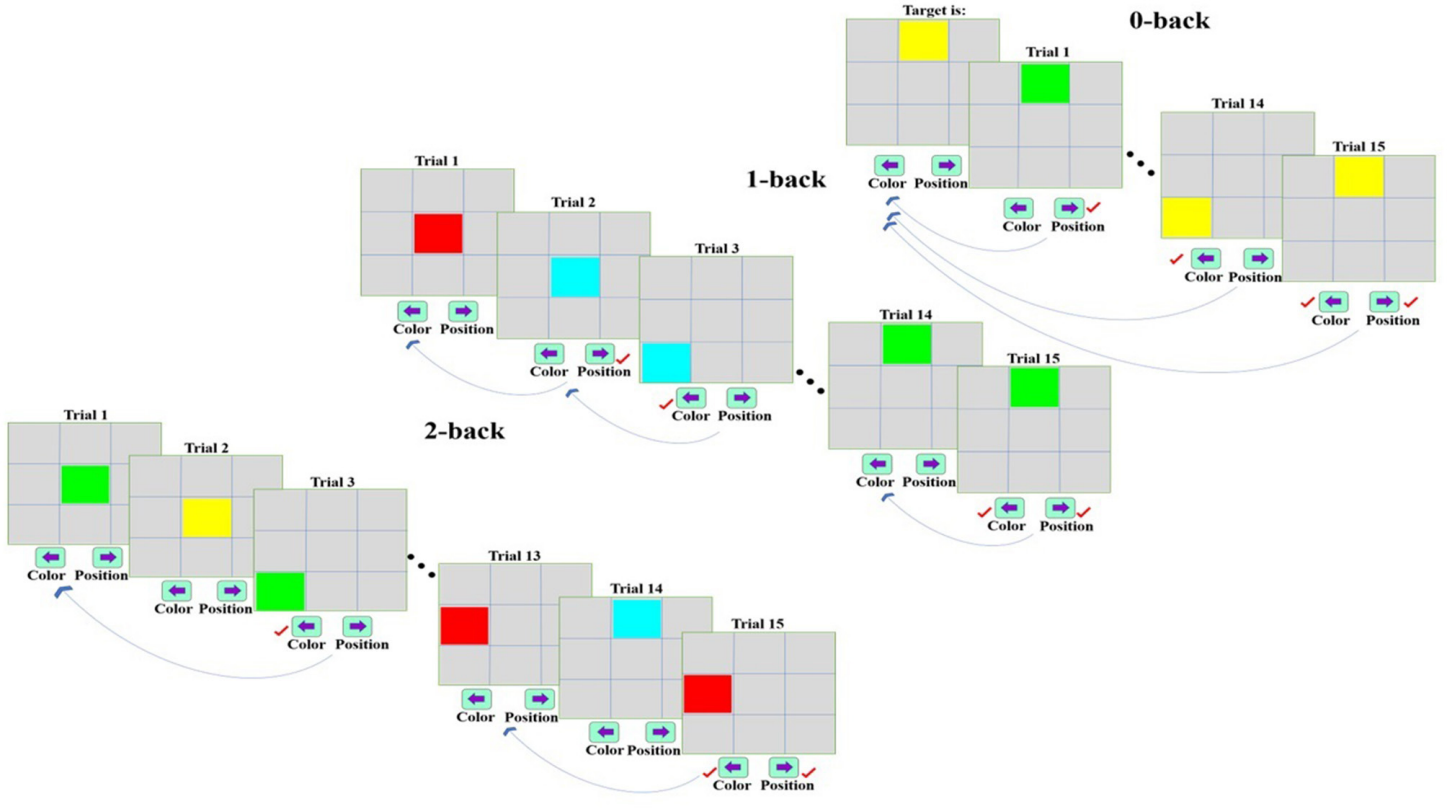
The dual n-back task with 0-, 1-, and 2-back levels [In the 0-back mode, a stimulus is presented as a target to the subject. After beginning the task, an individual should press the relevant key when each or both of color or location is similar to that of the target stimulus in each stimulation. The 1-back mode includes the comparison of each stimulus and the previous one concerning the correctness of color and location. Regarding the 2-back mode, each stimulus is compared with the stimulus in two prior trials in terms of color and location, and the relevant key is pressed if these stimuli match (Shirzadi et al., 2020)].

A dual n-back task was applied in the present study for examining working memory performance in males and females (Shirzadi et al., 2020). In the task, color and location were considered as target stimuli so that nine 3 × 3 square cells were in the middle of the gray screen. In each trial, one of nine square cells was displayed with one of the colors of red, green, blue, and yellow (RGBY), and an individual should remember the presented color and location of the stimuli, and respond according to each level of the task while observing target. The task included three difficulty levels (*n* = 0, 1, 2), and each of the memory load levels was repeated three times randomly during the task. In general, the task consisted of nine stimulation blocks, each of which involved 15 trials. Additionally, the display and interval time of stimuli were respectively set as 500 and 2,500 ms, and a 20-s rest time was considered between two consecutive stimuli blocks. After completing the 15-trial set, the instructions for the next block (which is randomly selected from 0, 1, and 2) are displayed for execution. Participants then perform the desired task according to those instructions. In the 0-back mode, one of the nine square cells with one of the RGBY colors was presented to the subject as the target stimulus. After starting the task, an individual should press the key related to each of the two targets simultaneously or immediately one after the other as a correct response in each stimulation when each or both color or location of the displayed cell matches the target stimulus. In this regard, the right and left arrow key was intended to respond to the correctness of target location and color, respectively. Regarding the 1-back stage, the keys corresponded to the correctness of the target should be pressed when the stimulus presented in each stimulation matches the previous stimulus in terms of color and location. The 2-back stage included comparing the stimulus displayed in each stimulation with that of two prior ones. The distance between subjects and screen was considered constant (0.5 m) and the task was performed in a quiet environment with low light. Figure 1 depicts the levels of the dual n-back task.

### 2.3 fNIRS data acquisition

**Figure 2:**
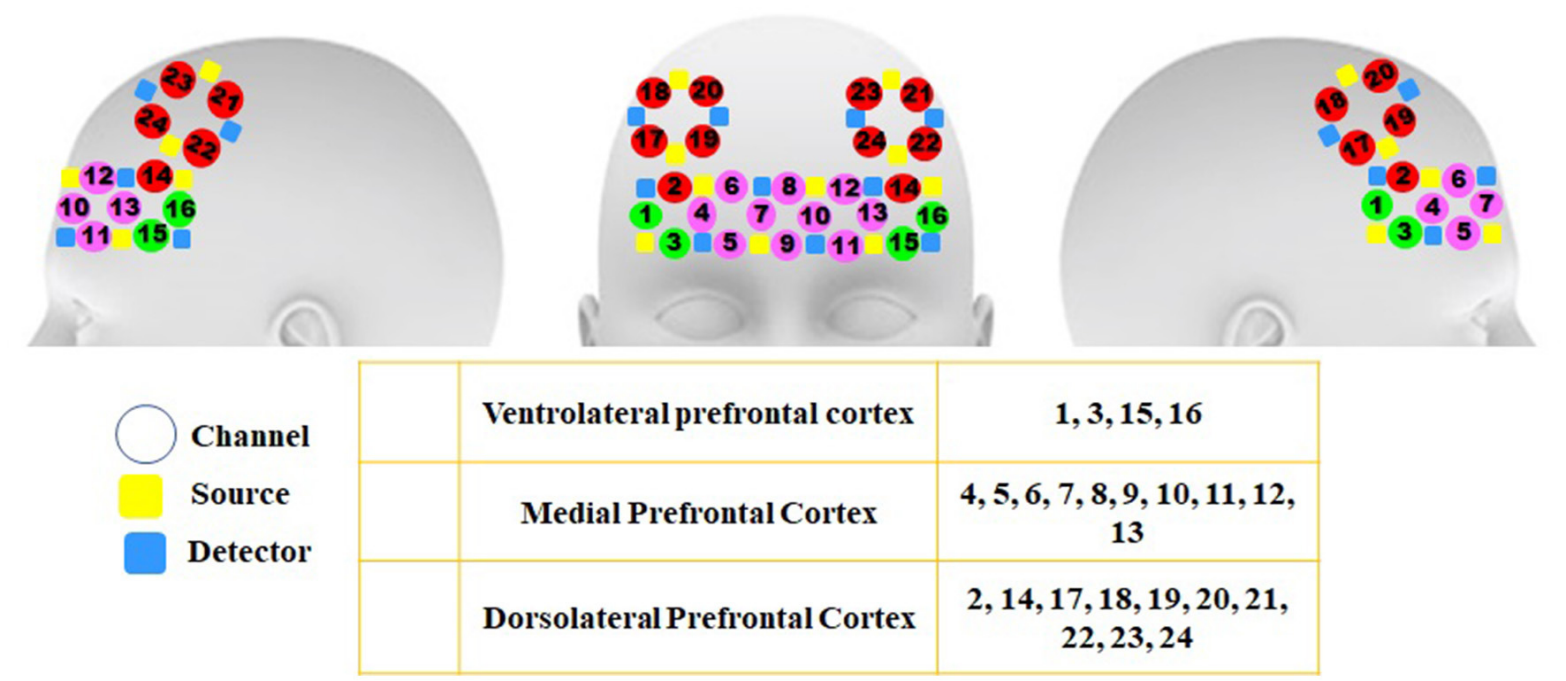
Arrangement of channels and the brain areas related to each channel in the frontal lobe These areas involve three regions of the frontal lobe in both hemispheres including channels 1, 3, 15, and 16 (VLPFC), 2, 14, and 17–24 (DLPFC), and 4–13 (MPFC).

A 48-channel fNIRS MR-compatible device (OxyMon fNIRS, Artinis) was utilized in the present study for recording the variations in the concentration of oxy- and deoxyhemoglobin. The device is located in the National Brain Mapping Laboratory (NBML) and could transmit infrared (IR) radiation at 730 and 850 nm, and 10-Hz sampling frequency, which can penetrate the skull and assess the brain cortex. Additionally, the changes in the concentration of blood oxy- and deoxyhemoglobin were calculated based on Beer-Lambert's law (Delpy et al., 1988; Obrig et al., 2000; Toronov et al., 2000; Boas et al., 2001). The results of the previous studies indicated that neuronal activity leads to consecutive changes in the concentrations of oxy- and deoxyhemoglobin. fNIRS signals were recorded from 24 channels involving 10 transmitters and 10 detectors located in the three different regions of the brain frontal cortex including ventrolateral (VLPFC), dorsolateral (DLPFC), and medial prefrontal cortex (MPFC). A 3-cm constant distance was considered between each transmitter and detector, which can evaluate the penetration depth of 1.5 cm. The arrangement of channels and their associated areas are provided in Figure 2.

### 2.4 Signal processing algorithm

**Figure 3:**
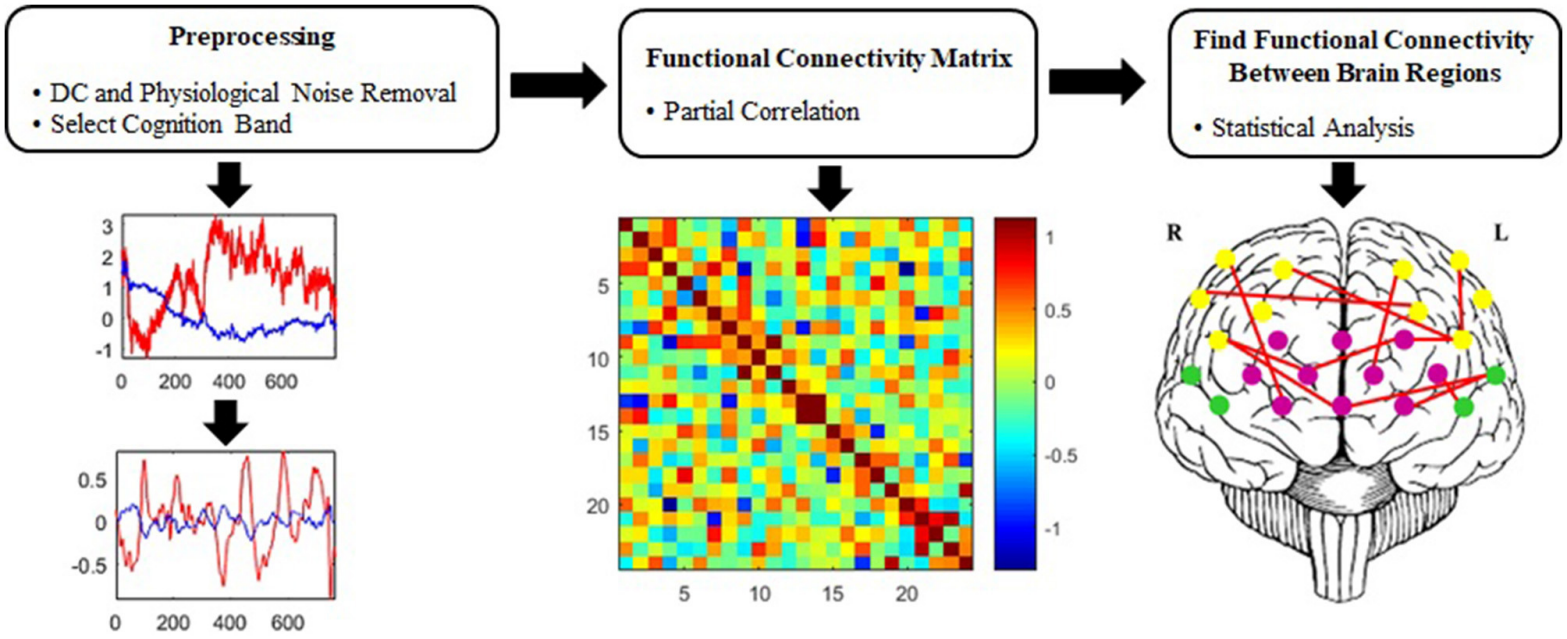
Proposed signal-processing algorithm.

Figure 3 represents the suggested signal-processing algorithm briefly. Wavelet transform is proposed for removing irrelevant physiological signals and artifacts (Emir et al., 2003; Supekar et al., 2008; Alonso et al., 2011; Skidmore et al., 2011; Molavi and Dumont, 2012; Zhang et al., 2014; Farahani et al., 2021), which was employed to extract an optimal cognitive band based on the frequency content of the fNIRS signal.

#### 2.4.1 Wavelet transform-based preprocessing

The different types of noises such as systemic (fluctuations in blood pressure, breathing, and heart rate) and motor artifacts are observed in the fNIRS signal. In general, systemic artifacts are associated with frequencies higher than 0.08 Hz, while neuronal activity-related signals occupy the frequency range of 0.003–0.08 Hz (Huppert et al., 2009; Sasai et al., 2011; Einalou et al., 2015, 2016, 2017; Dadgostar et al., 2016). Further, the noises of the fNIRS signal are eliminated by using a wavelet algorithm (Molavi and Dumont, 2012; Dadgostar et al., 2016). Discrete wavelet transform (DWT) uses filter banks for analyzing wavelet and decomposes the signal into the wavelet coefficients, which can present signal in different frequency bands. In the DWT, the coefficients of low- and high-pass filters are respectively named as an approximation (cA) and detail (cD). The selection of wavelet function plays an important role in the quality of analyzing fNIRS signals so that the similarity between mother wavelet and hemodynamic response function improves the detectability of the accuracy of estimating signal (Molavi and Dumont, 2012). Therefore, Daubechies 5 (Db5) was applied as a mother wavelet function for decomposition (Molavi and Dumont, 2012; Dadgostar et al., 2013, 2018). Considering the sampling frequency, the signal was decomposed into 11 levels, and the cA of the 11^th^ level was set to zero to remove the DC offset component from the signal (Dadgostar et al., 2016). Based on the wavelet decomposition tree, the detailed coefficients of the first to sixth levels were zeroed and the signal was reconstructed for each detector by using reverse wavelet transform to select the intended frequency band and eliminate physiological noises and high-frequency values (Dadgostar et al., 2016; Einalou et al., 2017; Shirzadi et al., 2020).

#### 2.4.2 Partial correlation

PC is considered a potent and useful tool for specifying the relationship between two variables after removing the effects of others (Marrelec et al., 2006). PC analysis aims at finding the latent relationship between two channels while eliminating the mutual effect of other channels. In the present study, 24 channels located in the different areas of the frontal lobe were utilized to assess brain functional connectivity. If *x* = (_*x*_*i*_)*i* = 1, …, *D*_ is assumed as the temporal signal related to each of 24 channels (*D* = 24) in the fNIRS signal, the partial correlation coefficient between channels i and j is defined by calculating their conditional correlation, regardless of the effect of other channels (*D*−2 channels remain, *R*\{(*i, j*)}), by as follows.


(1) $$\begin{array}{l} \underset{i,j}{\prod }=\text{corr}\left[x_{i},x_{j}|x_{R\backslash \left\{i,j\right\}}\right] \end{array}$$

PC matrix can be determined by computing the covariance matrix (Σ) of D channels. Thus, the number of the partial correlation coefficients obtained from the PC matrix ∏ = [_∏_*i, j*_]*D*×*D*_ is equal to *D*(*D*−1)/2 = 276 if *D* = 24. Also, the matrix ∏ can be determined by calculating the inverse of the covariance matrix ($\Upsilon =\left[\Upsilon_{i,j}\right]=\Sigma^{-1}$) (Dadgostar et al., 2016). Accordingly, we have:


(2) $$\begin{array}{l} \underset{i,j}{\prod }=-\frac{\Upsilon_{ij}}{\sqrt{\Upsilon_{ii}\Upsilon_{jj}}} \end{array}$$

where *i* and *j* demonstrate two separate channels and ∏_*ii*_ = 1. Further, PC values range between −1 and +1, which were computed for each frequency band in three types of stimulations (*n* = 0, 1, 2). Since PC analysis helps to eliminate the effect of indirect paths, it was employed to remove the PC between two channels associated with activity in other 22 areas (Marrelec et al., 2006; Hlinka et al., 2011; Dadgostar et al., 2016; Akin, 2017).

#### 2.4.3 Functional connectivity

Functional connectivity is defined as the temporal correlation between neurophysiological events in distant locations (Friston, 1994; Horwitz, 2003), which measures the simultaneous coupling between two-time series. Functional connectivity is often calculated between all elements in a system, regardless of whether the elements are directly connected structurally (Van Den Heuvel and Pol, 2010).

In the present study, functional connectivity matrices were determined for each individual in all three memory load levels. Therefore, three (24 × 24) symmetric matrices representing brain functional connectivity were obtained for each subject. Finally, the differences between partial correlation coefficients were investigated through two-way ANOVA statistical test at three stimulation levels (*n* = 0, 1, 2). We performed a two-way analysis of variance (ANOVA) test of HbO2 signals to investigate the specific effects of sex as well as possible sex × task interaction effects on brain functional connectivity during task performance over three cognitive workloads (*n* = 0, 1, 2) and two groups (males and females). “Tukey-kramer” method was performed for alpha correction. Two-way ANOVA model was defined with factors “sex” (male vs. female) and “task” (0-back vs. 1-back vs. 2-back).

**Figure 4:**
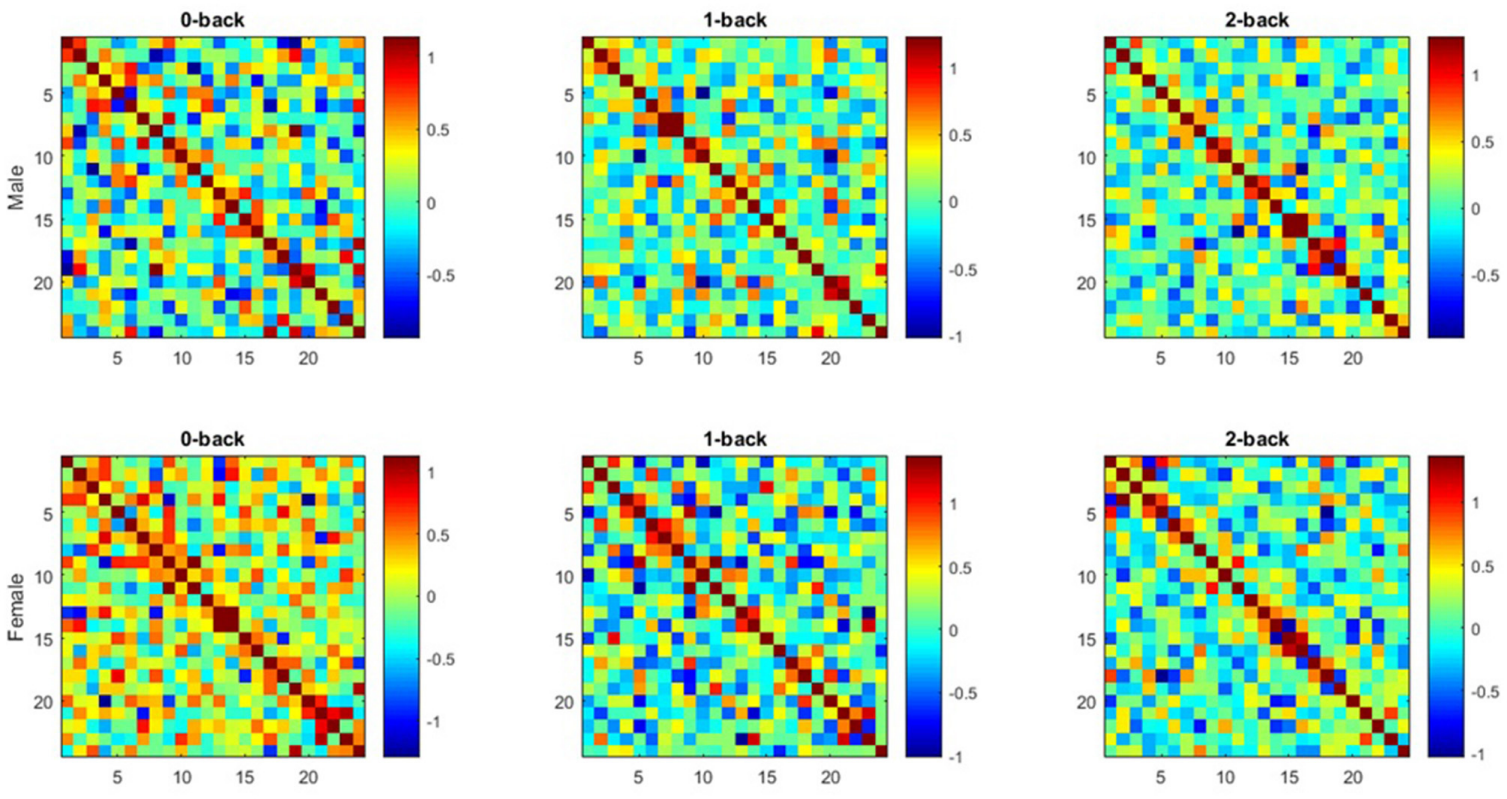
Average functional connectivity matrices extracted from both groups (Each Figure illustrates functional connectivity matrices for each channel pair of Hbo2 at all working memory load levels (0-, 1-, 2-back) in both groups as a 24 × 24 square matrix. x and y axes are considered as the channels mentioned in optode geometry, and each matrix unit indicates the mean functional connectivity strength between each channel pair).

The correlation matrices between the groups of males and females were extracted at all three memory workload levels by calculating the partial correlation coefficients between the hemodynamic responses of each channel pair in the frontal lobe (Figure 4). The colorbar represents the correlation strength which ranges between –1 and 1. Each cell of the matrix element demonstrates the mean functional connectivity strength between the corresponding channel pairs across all subjects.

## 3 Results

### 3.1 Behavioral results

To analyze behavioral results, the response time (RT) and accuracy rate (Acc) of the task were determined among 18 subjects for both target inputs (color and location stimuli) at three memory workload levels of the task (*n* = 0, 1, 2). Acc and RT were respectively calculated by averaging the correct responses to target stimuli, and their time in each of task levels and three memory workloads. There were not any outlier data, so there were not any outlier removal procedures. Table 2 summarizes the behavioral results for each target stimulus (color and location) in both sexes. In general, the behavioral results reflected that an increase in memory workload and task difficulty resulted in decreasing Acc and raising RT between both males and females.

**Table 2:** RT and Acc values related to color and location stimuli in each group of males and females.

**Behavioral data**				
	**Stimuli**			
	**Color**		**Location**	
**Group**	**Males**	**Females**	**Males**	**Females**
**Accuracy (Mean** ±**std)**				
0-back	0.64 ± 0.18	0.91 ± 0.13	0.55 ± 0.16	0.84 ± 0.15
1-back	0.57 ± 0.21	0.84 ± 0.12	0.44 ± 0.21	0.77 ± 0.25
2-back	0.35 ± 0.22	0.67 ± 0.27	0.28 ± 0.22	0.57 ± 0.22
**Reaction time (Mean** ±**std)**				
0-back	0.79 ± 0.27	0.71 ± 0.39	0.68 ± 0.06	0.93 ± 0.36
1-back	0.70 ± 0.49	1.18 ± 0.21	0.85 ± 0.17	1.13 ± 0.24
2-back	1.28 ± 0.29	1.64 ± 0.17	1.08 ± 0.1	1.65 ± 0.49

Based on comparing the results of RT to color stimulus among sexes, females responded slower than males in all modes except 0-back one. The response speed of males to location stimulus is higher compared to the females in all task modes. Regarding the results related to Acc in both stimuli, females represented greater Acc at all task levels compared to males.

RT and Acc were evaluated by using a two-way ANOVA for both stimuli between males and females at task load, sex and interaction between task load and sex. The results are provided in Table 3. The repeated measures ANOVA for RT of color stimuli indicated significant main effects of task load $\left[F_{\left(2,48\right)}=23.49,P<0.0001,\eta_{p}^{2}=0.4946\right]$, sex $\left[F_{\left(1,48\right)}=8.37,P=0.0057,\eta_{p}^{2}=0.1485\right]$ and sex × task load interaction $\left[F_{\left(2,48\right)}=3.84,P=0.0284,\eta_{p}^{2}=0.1379\right]$. For location stimuli, the RT ANOVA showed significant main effects of task load $\left[F_{\left(2,48\right)}=18.65,P<0.0001,\eta_{p}^{2}=0.4373\right]$ and sex $\left(F_{\left(1,48\right)}=22.85,P<0.0001,\eta_{p}^{2}=0.3225\right]$ but did not indicate a significant main effect of sex × task load interaction $\left[F_{\left(2,48\right)}=1.73,P=0.1875,\eta_{p}^{2}=0.0674\right]$. The repeated measures ANOVA for accuracy of color stimuli demonstrated significant main effects of task load $\left[F_{\left(2,48\right)}=8.48,P<0.0001,\eta_{p}^{2}=0.2612\right]$ and sex $\left[F_{\left(1,48\right)}=28.55,P<0.0001,\eta_{p}^{2}=0.3729\right]$ but did not show a significant main effect of sex × task load interaction $\left[F_{\left(2,48\right)}=0.08,P=0.924,\eta_{p}^{2}=0.0033\right]$. The accuracy ANOVA for location stimuli indicated significant effects of task load $\left[F_{\left(2,48\right)}=8.47,P<0.0001,\eta_{p}^{2}=0.2607\right]$ and sex $\left[F_{\left(1,48\right)}=29.94,P<0.0001,\eta_{p}^{2}=0.3842\right]$, but no significant effect of sex × task load interaction $\left[F_{\left(2,48\right)}=0.07,P=0.930,\eta_{p}^{2}=0.0030\right]$ was found.

**Table 3:** RT and Acc values related to color and location stimuli (*P* < 0.05 is illustrated as bold).

**Behavioral result: two-way ANOVA**								
	**RT**				**Acc**			
**Stimuli**	**Color**		**Location**		**Color**		**Location**	
	*F*	*P*	*F*	*P*	*F*	*P*	*F*	*P*
P (Task)	23.49	< 0.0001	18.65	< 0.0001	8.48	< 0.001	8.47	< 0.001
P (Sex)	8.37	0.0057	22.85	< 0.0001	28.55	< 0.0001	29.94	< 0.0001
P (Interaction)	3.84	**0.028**	1.73	0.187	0.08	0.924	0.07	0.930

**Figure 5:**
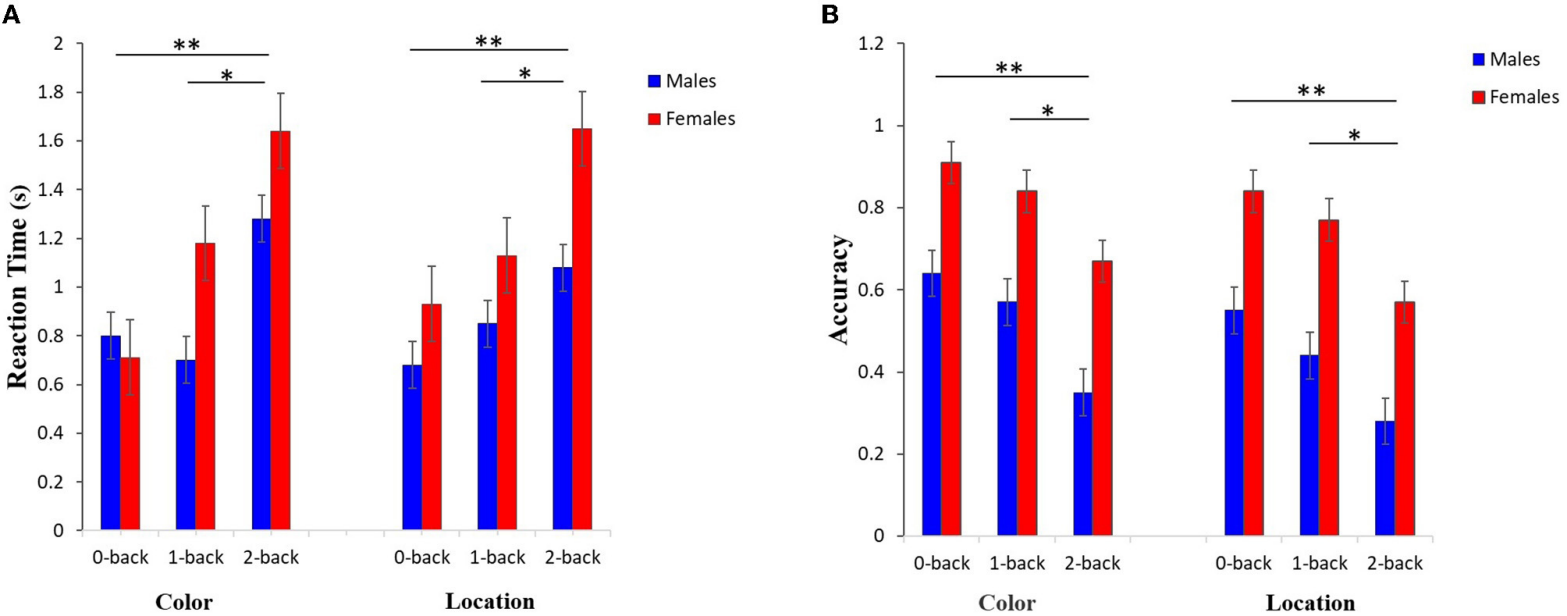
Results related to the *post-hoc* analysis of **(A)** RT and **(B)** Acc values for both input stimuli and three memory workload levels among males and females groups. The results reflect a significant difference between the working memory load levels of 0- and 2-back, as well as 1- and 2-back (^**^P < 0.001,^*^P < 0.05).

The *post-hoc* analysis indicated that for both RT and Acc there were significant effects among 0-back and 2-back (*P* < 0.001), as well as among 1-back and 2-back (RT-color *P* < 0.0001, RT-location: *P* < 0.001, Acc-color: *P* = 0.0141, Acc-location: *P* = 0.0251) in both color and location stimuli, but did not show a significant effect among 0-back and 1-back for sex in both stimuli (Figure 5).

### 3.2 fNIRS results

The results of functional connectivity on stored signals were determined in males and females by using partial correlation coefficient. The results were analyzed using two-way repeated measures ANOVA, the effects of sex and working memory loads on functional connectivity were examined simultaneously. The *post-hoc* analysis was used for multiple comparisons. Before applying the ANOVA test, Fisher's Z Transformation was utilized for normalizing data distribution.

The two-way ANOVA with the variables of sex (males vs. females) and working memory load (0- vs. 1- vs. 2-back) was employed to examine their interaction on the functional connectivity of various regions in the brain, the results of which are presented in Table 4. As shown, the significant interaction of sex × task loads is found in the connectivity between the nine channel pairs in the following regions.

**Table 4:** Results of two-way ANOVA among males and females groups (*P* < 0.05 is illustrated as bold).

**Two-way ANOVA: functional connectivity between channel pairs**							
	**Task**		**Sex**		**Interaction (Task** × **Sex)**		**Partial Eta-Squared**
	*F*	*p*	*F*	*p*	*F*	*p*	$\eta_{p}^{2}$
				**Channel pairs**			
4, 20	1.58	0.216	3.2	0.079	4.49	**0.016**	0.1575
5, 18	3.94	**0.026**	0.24	0.623	4.79	**0.012**	0.1665
10, 23	0.12	0.886	2.45	0.124	3.47	**0.039**	0.1262
11, 16	1.16	0.321	0	0.954	3.71	**0.032**	0.1338
12, 14	0.27	0.762	0.54	0.465	3.72	**0.031**	0.1341
14, 20	0.06	0.937	0.03	0.859	4.42	**0.017**	0.1556
14, 21	3.66	**0.033**	2.01	0.162	6.89	**0.002**	0.2231
14, 23	3.16	0.051	3.86	0.055	3.80	**0.029**	0.1368
17, 24	1.17	0.319	10.66	**0.002**	6.68	**0.002**	0.2178

Additionally, the results of two-way ANOVA indicated that the channel pairs of [5^*th*^, 18^*th*^:*F*_(2, 48)_ = 3.94, *P* = 0.026, $\eta_{p}^{2}=0.1410$] and [14^*th*^, 21^*st*^: *F*_(2, 48)_ = 3.66, *P* = 0.033, $\eta_{p}^{2}=0.1324$] are significantly different at task load, while no significant difference was observed between sexes. The channel pair (17^*th*^, 24^*rd*^) represented a significant difference among the sex [*F*_(1, 48)_ = 10.66, *P* = 0.002, $\eta_{p}^{2}=0.1818$]. However, a marginal difference was detected between channel pair (14^*th*^, 23^*rd*^) at task load [*F*_(2, 48)_ = 3.16, *P* = 0.051, $\eta_{p}^{2}=0.1163$], as well as sex [*F*_(1, 48)_ = 3.86, *P* = 0.055, $\eta_{p}^{2}=0.0744$].

The *post-hoc* analysis between sex and working memory load levels demonstrated significant effects in the channel pairs (5, 18), (14, 21), and (14, 23) in the regions of (R_MPFC, R_DLPFC), (L_DLPFC, L_DLPFC), and (L_DLPFC, L_DLPFC), respectively (Table 5). However, no significant effect was obtained in the other channel pairs. In the channel pair (5, 18), the mean of both groups reduced from the memory load level of 0- to 2-back (Mean: 0.953, *P*:0.029). Further, the levels of 0- and 1-back, as well as 1- and 2-back were not significantly different in terms of the mean. The mean in the channel pair (14, 21) was higher at 2-back level than the 0-back in both sexes (Mean: –0.754, *P*:0.025), while the levels of 0- and 1-back, as well as 1- and 2-back did not differ significantly. Regarding the channel pair (14, 23), the mean was greater in 1-back level compared to the 0-back (Mean: –0.683, *P*:0.041) although no significant difference was found among the other levels.

**Table 5:** Results of *post-hoc* analysis (*P* < 0.05 is illustrated as bold).

***Post-hoc*** **comparison**					
**Channel Pairs**	**Level (I) vs. Level (J)**	**Mean difference**	***P*** **-value**	95% **Confidence interval**	
				**Lower bound**	**Upper bound**
	0-back vs. 1-back	0.186	0.863	–0.684	1.057
5, 18	0-back vs. 2-back	0.953	**0.029**	0.082	1.824
	1-back vs. 2-back	0.767	0.094	–0.103	1.638
	0-back vs. 1-back	–0.426	0.287	–1.101	0.249
14, 21	0-back vs. 2-back	–0.753	**0.025**	–1.429	–0.078
	1-back vs. 2-back	–0.327	0.474	–1.002	0.347
	0-back vs. 1-back	–0.682	**0.040**	–1.341	–0.024
14, 23	0-back vs. 2-back	–0.297	0.523	–0.955	0.361
	1-back vs. 2-back	0.385	0.341	–0.273	1.043

In general, comparing the mean partial correlation between two groups of males and females, following an increase in the level of memory workload, indicated an inverse pattern in the pair of functional connectivity channels located in the right and left hemispheres, as well as in between-hemisphere connectivity in males toward females.

**Figure 6:**
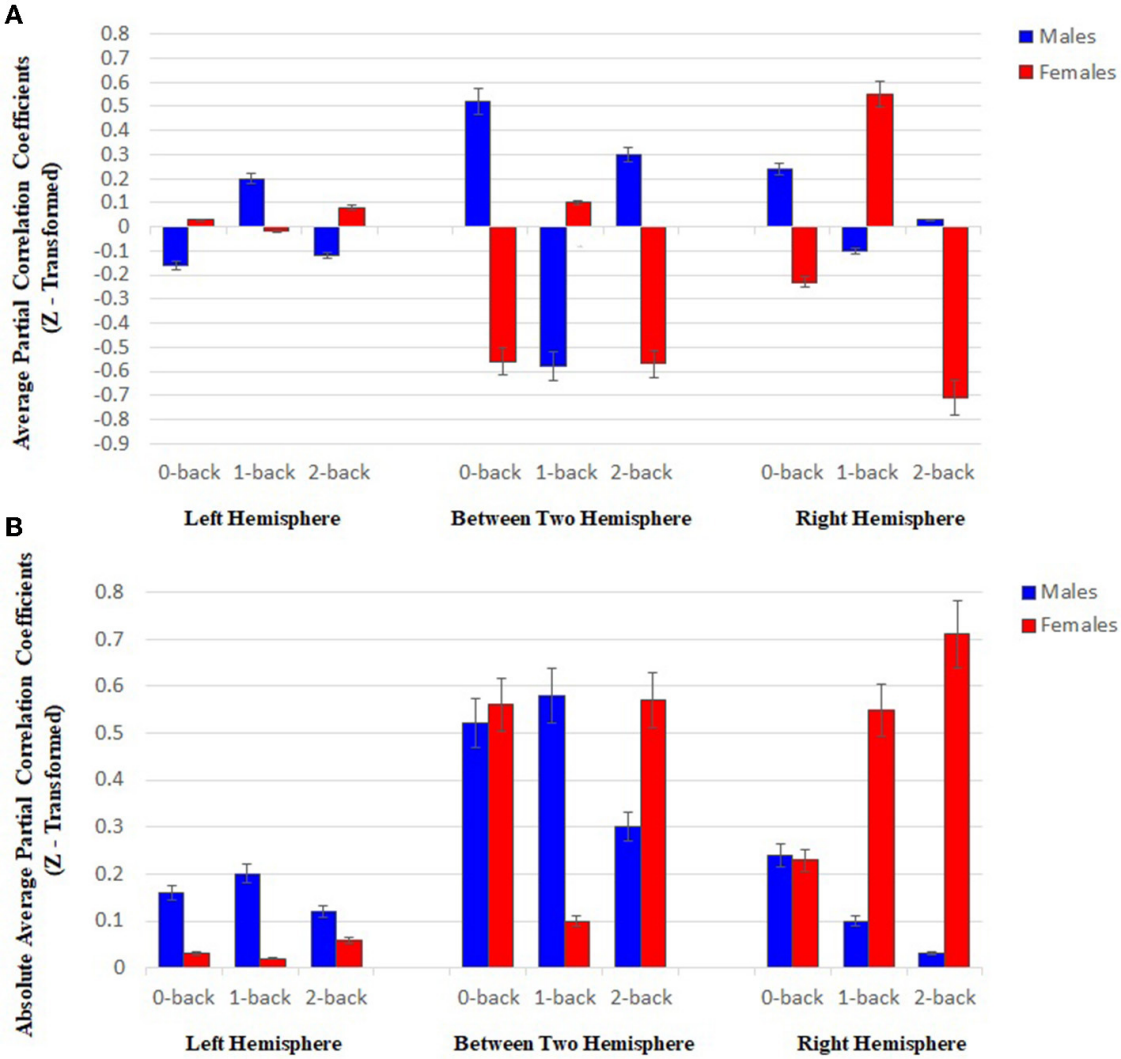
**(A)** Results of statistical analysis through using two-way ANOVA (*P* < 0.05), the mean partial correlation at three memory workload levels (0-, 1-, and 2-back) in the right and left hemispheres, and between-hemisphere connectivity (Each bar plot indicates the mean connectivity strength in two sexes at all three memory loads). **(B)** Results of statistical analysis through using two-way ANOVA (*P* < 0.05), the mean related to the sum of the absolute value of the partial correlation of the HbO2 signals in both groups, between the channels with functional connectivity at three memory workload levels (0-, 1-, and 2-back) in the right and left hemispheres, and between-hemisphere connectivity (Each plot bar represents the absolute value of mean connectivity strength in two groups at all three memory loads).

As shown in Figure 6A, the mean partial correlation in the left hemisphere of males and females is negative and positive in both 0- and 2-back levels, respectively. However, a positive and a negative mean are respectively observed among males and females in the 1-back. In addition, the means related to the channel pairs in the right hemisphere and between-hemisphere regions of males and females are respectively obtained positive and negative at both 0- and 2-back levels, while they are negative and positive in the 1-back, respectively.

The results of assessing each group separately reveal the inverse behavior of two hemispheres at different working memory load levels in each group. In the case of males, an increase was found in the mean partial correlation in the left hemisphere by enhancing brain working load from 0- to 1-back level, followed by declining the mean up to 2-back level. However, an inverse trend was detected in the right hemisphere. Regarding females, the mean related to the left hemisphere decreased from 0- to 1-back levels, and then promoted until 2-back, while the patterns were reversed in the right hemisphere.

As shown in Figure 6B, in the left hemisphere, the mean related to the sum of the absolute value of partial correlation at three memory workload levels in males was more than females (approximately 4-fold), while the mean value was higher in the females' right hemisphere than males (approximately 4-fold). The result reflected that males and females had stronger connectivity in the left and right hemispheres, respectively. Based on the results in Figure 6B, the channel pairs with significant connectivity in the right hemisphere exhibited an inverse pattern for males and females by raising memory workload level. In this hemisphere, an enhancement in memory workload led to a reducing trend in males and an increasing one in females.

## 4 Discussion

The present study aimed to evaluate the sex differences in frontal lobe functional connectivity during a dual working memory task. In general, the concepts of brain functional connectivity and activity play an important role in understanding brain performance, the first of which provides a deeper insight into the neural mechanism, as well as how the different brain areas interact (Medvedev et al., 2011; Zhang et al., 2014). Often, functional connectivity is computationally represented with the statistical dependence of a signal time series between separate brain areas. Numerous studies have focused on examining the task-invoked functional connectivity or that in resting-state under health and disease conditions (Fox and Greicius, 2010). Also, a large body of research has reported the sex differences in brain functional connectivity (Kilpatrick et al., 2006; Butler et al., 2007; Lv et al., 2010). The present study assessed brain functional connectivity by using partial correlation coefficient on fNIRS signals. PC measures the statistical connectivity of two regions based on the correlation condition between each pair of areas after removing the effects of other regions. Further, it is considered a potent tool for estimating brain functional connectivity, which is consistent with the results of the previous studies (Marrelec et al., 2007; Fransson and Marrelec, 2008; Ryali et al., 2012; Dadgostar et al., 2016). In the present study, a dual n-back working memory task with three difficulty levels (*n* = 0, 1, 2) was employed, in which two stimuli of color and location were considered as target inputs. Additionally, the oxyhemoglobin signals related to all three task levels were recorded. Due to the effectiveness of physiological system performance on oxyhemoglobin signals during neural activities, the signals were filtered by using DWT in the frequency band range of 0.003–0.08 Hz to decrease the effects of physiological fluctuations and device noise and extract the cognitive band of the brain (Einalou et al., 2014; Dadgostar et al., 2016). After extracting the functional connectivity matrix by using partial correlation coefficient, the connectivity between different frontal lobe areas was obtained through ANOVA. The results indicated that functional connectivity was higher in one of the hemispheres for each sex and the predominant hemisphere varied between males and females. While females exhibited stronger connectivity in the right hemisphere of the brain, this functional connectivity was stronger for males in the left hemisphere. Furthermore, an inverse pattern was observed between the average partial correlation between two groups of males and females with an increase in working memory load. The results also showed that the average absolute values of partial correlation in females increased with an increase in cognitive load and task difficulty, but a decrease pattern was observed in males.

Despite more than a century of scientific research, little advance has been made on the grounds of sex differences, especially regarding working memory. The previous studies evaluated the sex differences in brain connectivity and activation, and behavioral levels through various tasks such as Corsi-Block Tapping, item recognition, Sternberg, and n-back in different working memory domains (verbal and spatial). The literature on the sex differences in working memory performance and relevant brain activation is relatively rare and sometimes contradictory. Some researchers found no sex effect in the performance of working memory tasks (Bell et al., 2006; Schmidt et al., 2009; Li et al., 2010), while some others referred to the differences among the sexes (Speck et al., 2000; Lejbak et al., 2011).

In fact, the present study was pioneered in utilizing fNIRS signals for examining the sex differences in the functional connectivity between various frontal lobe regions during dual n-back working memory task.

### 4.1 Behavioral perspective

Based on the behavioral results of the present study, females represented fewer errors and greater ACC compared to the males despite spending slightly more time responding to both target stimuli (color and location) and had better performance in both color and location memories, which are consistent with those obtained in the previous research. The results of several studies reflected that females are superior in the spatial location-related memory and spatial WM tasks, while males possess better performance in navigational and rotational spatial tasks (Silverman and Eals, 1992; Eals and Silverman, 1994; Duff and Hampson, 2001). In the early 1990s, some researchers referred to females' superiority in the tasks needing location memory (Silverman and Eals, 1992; Sharps et al., 1993). Silverman and Eals (1992) provided an object identity memory task as a test for location-independent memory. Then, they presented conventional object location memory task and found that females perform in both tasks better than males. These results were repeated several times by different researchers using conventional object location memory task. The results of a review study by Voyer et al. (2007) demonstrated the significant sex differences in object location memory, which are in favor of females in most cases. The results indirectly emphasized that object identity and location are considered as a part of object location memory because of creating the sex differences in favor of females. McBurney et al. (1997) designed and implemented a memory game, named as object location task, and performed a mental rotation task among males and females subjects. They reported that the performance of males was significantly greater in mental rotation task, while females were significantly better in object memory, as well as remembering its location. Duff and Hampson (2001) evaluated sex differences by using spatial WM tasks and pointed out that females make significantly lower errors and represent better performance in the task. Particularly, females are more efficient in remembering the paths of locations by using a constantly-updated record of spatial information in tasks. Indeed, their results directly referred to the sex differences in the working memory of adult humans, which were confirmed by those obtained in the other studies (Voyer et al., 2007; Lejbak et al., 2009). Considering the behavioral results related to location stimulus in the present study, ACC was higher in females compared to the males, which demonstrates that females have more accuracy during responding despite their greater RT to stimuli. The results of other studies indicated the superiority of females in spatial WM tasks (Duff and Hampson, 2001; Harness et al., 2008). These experimental results reflected the fewer errors of females in spatial WM tasks compared to the males. For example, females seem to be better in perceptual speed and accuracy in visual tasks (Born et al., 1987; Duff and Hampson, 2001). In other words, females may perceive visual details faster and encode locations because of associating spatial WM to VSSP, as a working memory storage component. Consequently, they may understand and encrypt information location better (Duff and Hampson, 2001). Based on the results of the present study, females were superior in performing the task related to target location memory, which is in line with those of several studies. In comparison with males, females with less and stronger functional connectivity utilized the brain regions related to task more efficiently (Neubauer et al., 2002).

### 4.2 Cognitive perspective

Regarding the evaluation of brain performance through various brain imaging techniques, a small number of neuroimaging studies have specifically assessed the effect of sex on brain functional connectivity and activation during working memory tasks so far. Regarding brain activation, some researchers found similar lateralization patterns among the sexes (Haut and Barch, 2006; Schmidt et al., 2009), while some others supported more left and right hemisphere lateralization in females and males, respectively (Speck et al., 2000; Bell et al., 2006). However, the results of some studies indicated bilateral and left-sided PFC activation in females and males, respectively (Grabner et al., 2004; Chuang and Sun, 2014). The previous studies reported the activity and functional connectivity of various cortical regions in the frontal lobe during the n-back working memory task (Owen et al., 2005; Fishburn et al., 2014), which is in line with the result of the present study which reflects the connectivity between MPFC, DLPFC, and VLPFC areas in both hemispheres among males and females during the dual n-back task.

In the present study, the results of functional connectivity in both groups of males and females reflected that an increase in the task memory load led to a change and difference in the connectivity among different frontal lobe areas, as well as the strength of intra- and between-hemisphere connectivity. Following an increase in working memory load level, the mean partial correlation of two groups reflected an inverse behavior in the channel pairs having functional connectivity in the right and left hemispheres (Figure 6A). Further, this inverse pattern was found in between-hemisphere connectivity. In the left hemisphere, males exhibited stronger connectivity compared to the females, while stronger connectivity was observed in the females' right hemisphere (Figure 6B). Furthermore, an inverse trend was detected in the channel pairs with significant connectivity in the right hemisphere of males (falling) and females (rising) by enhancing working memory load level. The differences in the functional connectivity strength of males and females in two hemispheres may represent that two sexes apply various methods for encoding, processing, and retrieving memory at the diverse levels of task difficulty.

In general, the previous working memory tasks were examined in verbal and spatial domains. Based on the results of clinical and experimental studies, verbal WM tasks refer to left hemisphere lateralization due to the higher dependency of language production and perception on the hemisphere and the important role of this hemisphere in verbal processing. The results of different studies in the spatial domain demonstrated that spatial WM tasks use both brain hemispheres with more dominance of the right (McCarthy et al., 1994; Baldo and Shimamura, 2000; Shirzadi et al., 2020). The results are in agreement with those obtained in the present study, which reflect functional connectivity in the internal areas of the right hemisphere and between-hemisphere connectivity in both sexes. Also, neuroimaging studies reported bilateral involvement in verbal and spatial performances with a slight difference in the brain regions paying a role in each of the performances (Knauff et al., 2002; Goel and Dolan, 2004).

As already mentioned, various brain areas in both hemispheres were connected during the task in males and females, which is in line with the results of the past studies, which exhibit the involvement of two hemispheres in performing spatial WM tasks. A study was conducted among patients with hemispheric damage during verbal WM and spatial WM tasks in 2000, the results of which indicated that the individuals having left-hemispheric damage perform both tasks poorly, while those with right hemispheric damage are only weak in spatial WM ones. Accordingly, the left hemisphere is considered essential for solving both verbal WM and spatial WM tasks despite the greater involvement of the right one in spatial processing (Langdon and Warrington, 2000; Wilson Jr, 2012). Based on the results of the present study, the strength of functional connectivity was greater in females compared to the males which represents that females have more efficient connectivity and males are likely to need the higher number of connectivity in the brain for attaining an optimal performance comparable with that of females. Gao et al. (2018) applied NIRS and EEG simultaneously to assess the sex-related brain networks during the verbal working memory Sternberg task. The results of NIRS activation, functional connectivity, and small-world networks, indicated that males require more brain activity and optimal brain networks for achieving performance similar to that of females, which demonstrated that females outperform males in verbal Sternberg tasks. Further, the results of mass univariate analyses (MUA) indicated a significant sex difference in beta and gamma bands, which are mainly in the encoding period, by indicating that males and females may adopt various strategies for encoding memory. Most studies on spatial domain focused on location performances, the results of which exhibited the involvement of the right hemisphere in spatial WM or location-matching tasks, and storage of location-related information in this brain hemisphere (Hartley and Speer, 2000). These results are consistent with the results of the present study, which represent the presence of functional connectivity in the hemisphere during a task in both groups of males and females. As shown in Figure 6B, both sexes have intrahemispheric connectivity in their right hemisphere although in females, the strength of the connectivity between different areas of the right hemisphere is greater compared to males. The results of a review study in 2003 (Jager and Postma, 2003) demonstrated the dominance of the right hemisphere in spatial processing tasks such as location-matching ones. Another study was performed by using PET during the spatial WM task, which referred to the higher activity in the right-sided PFC in remembering object location (Smith et al., 1996).

### 4.3 Concluding remarks and implications for future research

The combination of behavioral and functional results for two sexes demonstrated a better performance in females due to the more effective use of the brain. The weak performance of males in solving working memory tasks (Silverman and Eals, 1992; Eals and Silverman, 1994; McBurney et al., 1997; Duff and Hampson, 2001) may be related to their strategies, as well as utilizing specific neural grounds. Furthermore, males may not be able to encode the mental object memory template seeming to be related to frontal activation, similar to the females (Levin et al., 2005).

The recent studies reported the importance of sex despite the general impression that spatial capability is a performance of the right hemisphere. In the present study, females represented better performance in target color memory compared to the males. Woodworth and Wells (1911) pointed out for the first time that females name colors in order faster than males, which was repeated in the previous studies by using different tasks (Saucier et al., 2002). Based on the behavioral results in Table 2, females perform better in color memory and remembering compared to males. Few studies have focused on color memory in performing tasks to evaluate the differences in sexes although a large body of research has highlighted target color naming and matching among and females. According to Pérez-Carpinell et al. (1998), a significant sex-related difference is observed in the remembered color (*p* < 0.05). They addressed that females remember colors better and more accurately than males, which is in agreement with the result of the present study. Additionally, right and left hemisphere plays a role in color processing (Njemanze et al., 1992; Sasaki et al., 2007; Njemanze, 2008) and memory (Simmons et al., 2007; Slotnick, 2009), respectively. The results of the present study reflected the presence of functional connectivity in both hemispheres among the sexes. Further, males possessed significantly more and stronger functional connectivity in their left hemisphere compared to females. The previous fMRI studies reported that color memory activates the brain cortical areas related to color perception in the left fusiform gyrus (Simmons et al., 2007; Slotnick, 2009). Considering this issue, as well as the results of the past research exhibiting the superiority of females in the tasks related to color naming, recognition, and memory, the need of males for higher interaction and more connectivity in the left hemisphere can be the reason for the greater strength of connectivity in their hemisphere compared to the females in the presents study. In general, neuroimaging data supported the claim that certain brain regions can perform differently in males and females to represent identical behavioral responses, which seems to be true in working memory.

Based on the results of the present study, fNIRS can be employed to examine the males-females differences in brain performance more reliably with fewer limitations compared to the fMRI. Furthermore, evidence was presented on the significant sex differences in human brain connectivity during working memory-related tasks, which underlies the sex-related cognitive differences. Thus, care to sex must be given when designing experiments and interpreting the results of brain network connectivity in health and disease. These data provide a framework for future studies using functional connectivity methods to clarify the basis of the males-females differences in applying brain networks during working memory tasks. Also, the use of functional neuroimaging allows researchers to create more accurate models of sex differences in the specific cognitive domains, which enables the theories of neuroanatomical and neurofunctional differences to be tested experimentally.

Despite the importance of understanding the differences between males and females socially and scientifically, few studies have addressed their potential neurophysiological basis. Addressing these issues can enhance the understanding of the neural networks which may be considered as responsible for sex differences in working memory, along with leading to the development of appropriate cognitive programs or new strategy potentially, which can decrease the sex gap (Irwing and Lynn, 2005, 2006).

The difference in brain functional connectivity may contribute to differences in the prevalence of neurodevelopmental diseases. For example, autism spectrum disorder (ASD) is 5–10 times more prevalent in males than the females (Association, 2000) due to its association with reducing local connectivity (Tommerdahl et al., 2008). Regarding schizophrenia, the symptoms, onset age, and duration of the disease are different among males and females patients. The differences in brain connectivity may be due to the differences in the natural evolution of these disorders in each sex. In total, the results reflected the significant sex differences in the central organization of the human brain, which can explain the differences in behavior and emotions among males and females. The difference is related to the importance of sex chromosome genes in brain growth and performance (Disteche, 2006). Accordingly, paying attention to sex can be considered important during designing experiments or interpreting the results related to brain network connectivity under health and disease conditions.

The recent neuroimaging studies provided significant evidence that sex is different in brain connectivity. Thus, the significant effect of sex in the patterns of human brain neural connectivity is likely to underlie the cognitive and behavioral differences among males and female. Finally, the results of the present study strengthen the presence of sex effect in the functional imaging research of hemodynamic function and emphasize the importance of evaluating brain network connectivity to acquire a greater scientific understanding of male-female differences.

### 4.4 Limitation of the study

One of the limitations of this study was the small number of subjects under investigation. A better evaluation of the differences in male and female cognition in this regard requires more extensive studies with a larger statistical population. Moreover, since menstrual fluctuations are related to the gonadal hormones which are important in brain sex difference (McEwen, 2001), incapability to control the menstrual cycle in testing females is considered as another limitation of the present study. In our study, we have only examined the changes in brain connectivity during a two-dimensional working memory task. The effects of each of the task stimuli were not separately investigated via a one-dimensional working memory task. This limitation is among the constraints of our study. However, an individual analysis and comparison of each of the stimuli during a one-dimensional working memory task with the results of the two-dimensional task could potentially provide more robust and reliable results. Given that prefrontal cortex (PFC) is a crucial region that plays a significant role in working memory and can be influenced by gonadal hormones, we focused our study solely on this area of the brain. Therefore, to obtain a more accurate understanding of the differences in the brain functional connectivity between sexes, it would be possible to record the signal from all brain regions.

## 5 Conclusion

In this study, a two-dimensional spatial working memory task (dual n-back) , was utilized during fNIRS data collection from healthy right-handed males and females. A partial correlation analysis was employed to extract the functional connectivity of 24 channels, which are associated with 24 frontal regions. Our results suggested that by increasing task memory loads, functional connectivity in both males and females led to changes in the strength between frontal lobe regions. Females demonstrated greater functional connectivity within the right hemisphere, while males showed a higher strength of functional connectivity in the left hemisphere. In both sexes, an inverse trend was detected in the results of the mean partial correlation as memory load level increased. This was observed in the channel pairs having functional connectivity in two hemispheres. Also, based on the results of the mean related to the sum of the absolute value of partial correlation at three memory workload levels in the right hemisphere, males showed a falling pattern, while females reflected a rising pattern.

## Data availability statement

The raw data supporting the conclusions of this article will be made available by the authors, without undue reservation.

## Ethics statement

The studies involving humans were approved by Ethics Committee of the Iran University of Medical Sciences (Code: IR.IUMS.REC. 1397.161). The studies were conducted in accordance with the local legislation and institutional requirements. The participants provided their written informed consent to participate in this study.

## Author contributions

SS is the master student who performed all the data collection, analysis, and contributed to the writing of the paper. MD and ZE are the academic advisors who developed the experimental protocol, mentored in data analysis, and contributed to the writing of the paper. SE and AA are academic mentors who contributed to the data analysis review, interpretation of the results, and revisions to the paper. All authors contributed to the article and approved the submitted version.
